# Target Heterogeneity in Oncology: The Best Predictor for Differential Response to Radioligand Therapy in Neuroendocrine Tumors and Prostate Cancer

**DOI:** 10.3390/cancers13143607

**Published:** 2021-07-19

**Authors:** Ameya D Puranik, Clarisse Dromain, Neil Fleshner, Mike Sathekge, Marianne Pavel, Nina Eberhardt, Friedemann Zengerling, Ralf Marienfeld, Michael Grunert, Vikas Prasad

**Affiliations:** 1 Department of Nuclear Medicine and Molecular Imaging, Tata Memorial Hospital, Homi Bhabha National Institute, Mumbai 400012, India; ameya2812@gmail.com; 2 Department of Diagnostic and Interventional Radiology, CHUV University Hospital, Rue du Bugnon 46, 1011 Lausanne, Switzerland; Clarisse.Dromain@chuv.ch; 3 Department of Urology, University of Toronto, 610 University Avenue Toronto, Toronto, ON M5G 2M9, Canada; Neil.Fleshner@uhn.ca; 4 Department of Nuclear Medicine, University of Pretoria and Steve Biko Academic Hospital, Pretoria 0001, South Africa; mike.sathekge@up.ac.za; 5 Department of Medicine, Friedrich-Alexander University Erlangen-Nürnberg, 91054 Erlangen, Germany; Marianne.Pavel@uk-erlangen.de; 6 Department of Nuclear Medicine, University Hospital Ulm, 89081 Ulm, Germany; Nina.Eberhardt@uniklinik-ulm.de (N.E.); michael.grunert@uni-ulm.de (M.G.); 7 Department of Urology, University Hospital Ulm, 89081 Ulm, Germany; Friedemann.Zengerling@uniklinik-ulm.de; 8 Institute of Pathology, University Hospital Ulm, 89081 Ulm, Germany; Ralf.Marienfeld@unikliniulm.de; 9 Department of Nuclear Medicine, German Armed Forces Hospital of Ulm, 89081 Ulm, Germany

**Keywords:** heterogeneity, Prostate Cancer, neuroendocrine tumor, PSMA, Ga-68, PET/CT, theranostics

## Abstract

**Simple Summary:**

In the era of precision medicine, novel targets have emerged on the surface of cancer cells, which have been exploited for the purpose of radioligand therapy. However, there have been variations in the way these receptors are expressed, especially in prostate cancers and neuroendocrine tumors. This variable expression of receptors across the grades of cancers led to the concept of ‘target heterogeneity’, which has not just impacted therapeutic decisions but also their outcomes. Radiopharmaceuticals targeting receptors need to be used when there are specific indicators—either clinical, radiological, or at molecular level—warranting their use. In addition, response to these radioligands can be assessed using different techniques, whereby we can prognosticate further outcomes. We shall also discuss, in this review, the conventional as well as novel approaches of detecting heterogeneity in prostate cancers and neuroendocrine tumors.

**Abstract:**

Tumor or target heterogeneity (TH) implies presence of variable cellular populations having different genomic characteristics within the same tumor, or in different tumor sites of the same patient. The challenge is to identify this heterogeneity, as it has emerged as the most common cause of ‘treatment resistance’, to current therapeutic agents. We have focused our discussion on ‘Prostate Cancer’ and ‘Neuroendocrine Tumors’, and looked at the established methods for demonstrating heterogeneity, each with its advantages and drawbacks. Also, the available theranostic radiotracers targeting PSMA and somatostatin receptors combined with targeted systemic agents, have been described. Lu-177 labeled PSMA and DOTATATE are the ‘standard of care’ radionuclide therapeutic tracers for management of progressive treatment-resistant prostate cancer and NET. These approved therapies have shown reasonable benefit in treatment outcome, with improvement in quality of life parameters. Various biomarkers and predictors of response to radionuclide therapies targeting TH which are currently available and those which can be explored have been elaborated in details. Imaging-based features using artificial intelligence (AI) need to be developed to further predict the presence of TH. Also, novel theranostic tools binding to newer targets on surface of cancer cell should be explored to overcome the treatment resistance to current treatment regimens.

## 1. Introduction

At last gleams of light have come, and I am almost convinced that species are not immutable, C. Darwin (letter to Joseph Dalton Hooker (11 January 1844). What Charles Darwin wrote to Joseph Dalton Hooker holds a great degree of significance in the management of cancer patients. The transformation of normal cells of human beings into a cancerous lesion takes place over the course of several years. During this process of carcinogenesis, these cells adapt continuously to the inherent instinct of survival in adverse situations. Not surprisingly, they behave like a different species, and by the time they metastasize, they possess diverse dissimilar characters, termed as tumor heterogeneity. The reasons for heterogeneity of tumor clones amongst patients and within metastases can be several, guided primarily through activated and deactivated genes and genomes, either due to intrinsic programming or through external therapeutic pressure. This had led to a paradigm shift in the management of cancers i.e., from blanket treatment with ‘nonspecific’ chemotherapy to exploring treatment options for targeting cancer cells specifically and tailored according to the need of a patient (personalized medicine). Radioligand therapy (RLT) is one such promising treatment option, wherein therapeutic alpha, beta, or electron ray emitting radiopharmaceuticals bind to specific receptors or antigens on tumor cell surfaces, thereby producing direct tumoricidal action with minimal/manageable side effects and toxicity. The entire success of these therapies and their position in the current treatment algorithm of respective cancers depend on the principles of good patient selection, robust imaging biomarkers for response assessment, and its impact on quality of life parameters [1]. Preliminary clinical trials on RLT in different types of cancer have been successful and a number of clinical phase II and III trials are already being conducted to generate robust evidence for the same. NETTER-1 trial did establish PRRT with Lu-177 DOTATATE as ‘standard of care’ for management of metastatic or locally advanced well-differentiated neuroendocrine neoplasms (NENs) [2]. Likewise, PSMA RLT with Lu-177 PSMA is gaining firm ground in the diagnostic and therapeutic algorithm of prostate cancer (PC). Of late, there has been a resurgence in radioimmunotherapies, e.g., with Y-90-labeled anti-CD66 antibodies for bone marrow ablation prior to bone marrow transplantation for leukemic and myelodysplastic syndrome patients. Even the established RLTs such as I-131-MIBG and I-131 radioactive iodine have been offered a relook in light of this concept of tumor target heterogeneity. We, through this article, shall be exploring the evolution of the concept of tumor heterogeneity, its extrapolation to the principles of RLT, and the current biomarkers available to predict the success of RLTs in clinical practice today. As the current ‘standard of care’ for radioligand therapy is well-established in NET and prostate cancer, this review shall focus on these tumor types.

## 2. General Concept of Tumor Heterogeneity

### 2.1. Tumor Evolution and Its Non-Invasive Assessment

The biggest challenge in the treatment of cancers is the development of resistance to therapies. This ability of cancer to adapt to pharmacologic pressures can be described in terms of tumor evolution and stems from its intrinsic diversity or heterogeneity. Multi-region sequencing is one of the techniques to study tumor evolution, which involves parallel analysis of tissue derived from different regions of a single neoplastic mass, and from distinct metastatic lesions from the same patient [3]. Clonal alterations, present in all samples analyzed likely represent ‘ancestral’ events, occurred early in tumorigenesis, whereas the ‘heterogenous events’ that have occurred later are a result of the subclonal alterations [4]. Recently, the increase in sensitivity of DNA-sequencing techniques has allowed genetic characterization of tumors from the analysis of circulating tumor DNA (ctDNA) isolated from plasma and other biological fluids. Analysis of ctDNA is based on the identification of tumor-specific alterations, which accounts for its high specificity and sensitivity and detection rates comparable with those of tissue biopsies [5]. Liquid biopsy allows the tracking of the evolution of different cell subclones, and this was proven to be particularly effective in the follow-up of patients with prostate cancer treated with targeted therapy in the metastatic setting [6].

### 2.2. Target Heterogeneity in Cancers: Inter- and Intratumor Heterogeneity

Tumor or target heterogeneity refers to the coexistence of cellular populations bearing different genetic or epigenetic alterations within the same lesion, or in different lesions of the same patient. Intratumor heterogeneity is characterized by its dynamic changes. Tumor initiation and progression are generated from stochastic to sequential mutations that contribute to subsequent clonal expansion and intratumor heterogeneity [7]. Therefore, a single biopsy is unlikely to capture the complete genomic landscape of a patient’s tumor considering the spatiotemporal changes in tumor heterogeneity. Today, the knowledge and the clinical evaluation of tumor heterogeneity are extremely important to improve clinical oncology. Intertumor heterogeneity exceeds the boundaries of specific tumors and also of their molecular classifications [8,9], which makes the clinical approach very complex. However, the most complex issue is intratumor heterogeneity (ITH) as a spatial and temporal phenomenon more or less distinct in every single patient [10]. This is closely related with cancer progression, resistance to therapy, and recurrences. Because of ITH in primary tumors and metastases, and because of the wide clinical heterogeneity among patients, it is necessary to apply clinical research methods directly to patient material in today’s clinical practice to be able to better define a specific effective treatment.

### 2.3. Heterogeneity and Grading of Cancers

As the cancer transforms or evolves from a low grade to intermediate to high grade, the cell population undergoes transformation. This has been the most significant hallmark of cancers, which has led to development of grade-specific treatment strategies [11]. Well-differentiated NENs of intermediate grade (WHO Grade 2) demonstrate differential expression of somatostatin and glucose transporter receptors, which are indicators of co-existence of well and poorly differentiated components, respectively [12]. Similarly, prostate carcinogenesis and progression to an androgen-independent state are dependent on androgen receptor (AR) expression and function. Although somatic AR mutations are rarely detected in early-stage prostate cancer, mutation frequency is significantly increased in advanced androgen-independent tumors, suggesting that AR mutations have a role in tumor progression. The highly heterogeneous nature of prostate cancer provides a real challenge for clinical disease management, and it is becoming increasingly clear that patients from different geographical and ethnic backgrounds harbor different genomic alterations, suggesting that distinct pathways of prostate carcinogenesis exist [13]. 

## 3. Approaches to Assess Tumor Heterogeneity

As the grade of cancer progresses, the approach needs to be changed, and it is not often that a treating physician recognizes this grade progression. Often, it is the imaging-based progression that detects this or an increase in circulating biomarkers, and further provides a target for re-biopsy, following which there is pathological and molecular evidence of heterogeneity as a possible explanation for disease worsening. 

### 3.1. In Vitro Molecular Pathology

In the context of PRRT, Ki-67 remains the strongest predictor of prognosis, as has been demonstrated in patients with NET G1 and low G2 (3–10%) range showing significantly improved PFS and OS compared to higher proliferating tumors. Several previous studies have been constructed based on Ki-67 cutoff thresholds and NET grade was used for its prognostic impact as stratification factor. This has also entered recommendations of treatment strategies in international guidelines, e.g., the guidelines of European Neuroendocrine Tumor Society (ENETS) and the North American Neuroendocrine Tumors Society (NANETS) are mostly centered around Ki-67 [14,15]. Even though the Ki-67 index is most commonly used for grading NETs worldwide, it is subject to sampling error because the Ki-67 index is not uniform within the whole tumor or the metastases. 

### 3.2. Serum-Based Biomarkers

**Figure 1:**
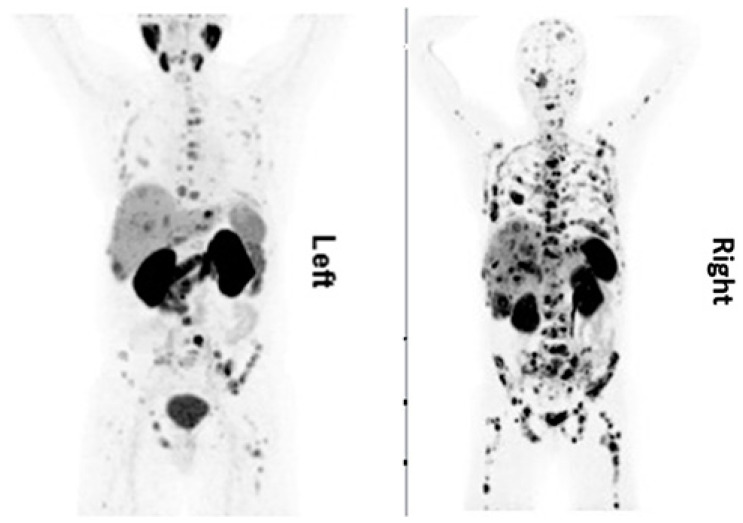
A 61-year-old patient, first diagnosed in 08/2015 with histological diagnosis of adenocarcinoma of prostate, Gleason score 5 + 4, was referred for radioligand therapy due to disease progression after treatment with firmagon, docetaxel, lucrin, external beam radiation therapy and 1 cycle of cisplatin/cabazitaxel. His lab values showed PSA < 0.01 µg/L, CgA 767 ng/mL, NSE 277 µg/L. PSMA PET/CT (**Left**) was performed to assess the feasibility of performing Lu-177 PSMA therapy. Because of his low PSA level and elevated tumor markers for neuroendocrine differentiation, this patient was also examined with Ga-68 DOTATOC PET/CT (**Right**). The maximum projection intensity images showed more somatostatin receptor positive lesions as compared to PSMA avid lesions. Images provided by Rachelle Steyn from University of Cape Town, South Africa.

Biomarkers include tools and technologies that can facilitate the diagnosis, cause, progression, or regression, prediction of treatment of disease or reflect tumor burden. In general, biological markers (biomarkers) are considered ‘cellular, biochemical or molecular alterations that are measurable in biological media such as human tissues, cells, or fluids’. The progress and challenges presented by these biomarkers assume a special relevance given the heterogeneity of the neoplasia and diverse anatomical and cellular origins. As a consequence, these tumors produce a wide range of measurable active and inactive products and reflect a broad spectrum of tumor biological behavior. In neuroendocrine tumors, plasma chromogranin A (CgA) is one of the most commonly evaluated biomarkers in patients with neuroendocrine tumors. The sensitivity is around 70 percent but highly variable depending on the used assays. The European Society for Medical Oncology (ESMO) and ENETS guidelines recommends complementing imaging procedures with plasma CgA measurement at baseline and for monitoring during treatment and follow-up of patients with GEP-NETs if elevated at baseline [16]. The prognostic value of CgA has been shown in multiple studies; however, the predictive value has been shown just for SSA therapy in some studies, while the PRRT response does not correlate with CgA level changes [17]. The use of plasma CgA levels is limited by elevation related to other diseases or conditions (non-NET malignancies, hepatic impairment, or renal insufficiency, intake of proton pump inhibitors [18]. Other monoanalyte biomarkers such as pancreastatin, neurokinin A (NKA), neuron-specific enolase (NSE), pre-progastrin, pancreatic polypeptide (PP), serotonin (5-HT), and urinary or plasma 5-HIAA also do not meet the accepted standards of care for diagnosis, staging, and follow-up of patients [19]. In prostate cancer, prostate-specific antigen has been used as the ‘standard of care’ marker for detection and prognostication of prostate cancer since the 1990s. The fact that the significant association between higher PSA and worse outcomes remains after years of clinical use suggests PSA remains a robust prognostic variable among men with newly diagnosed prostate cancer. However, lack of specificity and false positives have hampered its utility. Moreover, in scenarios where the patient becomes castrate resistant, PSA may not be relied upon, e.g., in patients with neuroendocrine differentiation prognostication, and in this regard, imaging biomarkers have a significant impact (see Figure 1). Androgen-deprivation therapies (ADT) are part of standard treatment algorithm for prostate cancers. ADT is effective initially but a majority of tumors relapse with castration-resistant prostate cancer (CRPC). CRPC is driven primarily by aberrant activation of AR in the milieu of castrate serum levels of androgen. On the other hand, approximately 25% of the men who die of prostate cancer have tumors with an associated neuroendocrine phenotype, which is also a harbinger of poor prognosis. There are multiple molecular processes in play during this transformation. ADT leads to activation of CREB (cAMP response element-binding protein) which, in turn, promotes neuroendocrine differentiation (NED) of prostate cancer cells [20]. In AR-positive prostate cancer cells, CREB-binding protein (CBP), a histone acetyltransferase, has been shown to act as an AR coactivator in transcriptional activation of AR target genes. In addition, ADT-activated CREB promotes angiogenesis and NED [21].

### 3.3. Molecular Imaging-Based Biomarkers

Even today, Krennings score has been used as a point of reference for selecting patients for PRRT [22]. The NETPET score devised by Chan et al. has given some objectivity for characterizing FDG- and Ga-68-PET-positive NETs; however it lacks prospective validation, especially from the point of view of prognostication [23]. Metabolic parameters such as SUV max, SUV mean, metabolic tumor volume (MTV), and total lesion glycolysis (TLG) have failed to provide any consistent results from the point of view of prognostication [24]. As we go up the ladder of differentiation, there emerges an inherent heterogeneity, with co-expression of SSTR and glucose-transporter (GLUT) receptors. Thus, addition of FDG PET imaging in the well-differentiated intermediate or high-grade NETs identifies the heterogeneous components of disease. It has been documented that addition of chemotherapy following ‘positive FDG PET’ study shows good response on short-term follow-up [25]. There still remains a significant subset of tumors which show absence of objective response (or stable disease) in spite of showing high SSTR expression, low Ki-67, low liver tumor burden, and absence of FDG uptake. Graf et al. have proposed that amongst all the known relevant clinical and pathological parameters, the ‘quality’ of SSTR expression, assessed visually on PET/CT, as a significant predictive as well as prognostic parameter [26]. Patient selection based on visual SSTR expression on a maximum intensity projection (MIP) image has been the accepted reference standard, though does not account for the differential SSTR expression within the lesions. The short range of lutetium-177 can miss the volume of tumor which has low SSTR expression and is beyond its range; it is very likely that these components of the lesion or tumor progress faster although the other sites or lesions attain good response. Hence, the quality of SSTR expression was visually assessed as follows: lesions greater than 2 cm and definitely visible on CT (target lesions) were considered for the assessment. The variability of SSTR expression over three planes was analyzed, and patients were attributed to having heterogeneous SSTR expression if ≥50% of their lesions were heterogeneous. Median time-to-progression of patients with heterogeneous SSTR expression, containing both grade 1 and grade 2 NET was 28 months and as such shorter than in patients with homogeneous SSTR expression. Thus ‘quality’ of SSTR expression has emerged as another independent parameter for predicting response to PRRT. A similar conundrum is faced when functional imaging is used in prostate cancer. Typically, patients with exhausted conventional therapy options are selected based on PSMA overexpression (variably defined) as seen on a PSMA positron emission tomography (PET) scan. However, as Current et al. also point out, a significant proportion of patients do not respond to PSMA RLT despite using the theranostic approach [27]. Therefore, optimal patient selection is of paramount importance for rationalization of PSMA RLT as treatment failures are seen in roughly 30% which needs to be identified. This is routinely done using an imaging-based approach. PSMA PET/CT helps in identifying lesions with high PSMA expression, as the same receptor is targeted with therapeutic radiotracers by the principle of theranostics. In a study by Ravikumar et al. [28], a subset of treated patient cohort underwent dosimetry, wherein 1 of 11 patients with a whole-body tumor that absorbed a dose under 10 Gy had a PSA response greater than 50%. As part of their phase 2 ^177^Lu-PSMA trial, Hofman et al. [29] performed FDG PET/CT alongside 68Ga-PSMA-11 PET/CT at baseline to characterize imaging phenotypes and select patients who were best suited for Lu-177-PSMA-617 therapy. Sixteen patients (median age 71 yr, range 58–88) who were screened for the trial were excluded on the basis of low PSMA expression or discordant FDG-avid disease. Fifteen of 16 patients died during the follow-up period, with a median OS of 2.5 months. Subgroup analysis for patients with low PSMA expression or discordant FDG-avid disease showed median OS of 2.3 and 3.9 months, respectively [30]. Thus, tracer concentration of PSMA and FDG PET imaging has the potential to become an important prognostic parameter to guide further management. 

Schmidkonz et al. [31], in a study of 142 patients, performed a quantitative assessment of all 641 PSMA-positive lesions in the field of view to calculate PSMA-derived parameters, including whole-body PSMA tumor volume (PSMA-TV) and whole-body total lesion PSMA (TL-PSMA) as well as the established SUVmax and SUVmean values. All PET-derived parameters were tested for correlation with serum PSA levels and for association with Gleason scores. PSMA-TV and TL-PSMA demonstrated a significant correlation with serum PSA levels. Interestingly, TL-PSMA was the only PET-derived parameter which was significantly different between patient groups with different Gleason scores. Additionally, response-to-therapy assessment using TL-PSMA showed the highest agreement to monitoring based on PSA levels, superior to SUVmax-based evaluation and response assessment based on CT data and RECIST 1.1 criteria [31].

### 3.4. Liquid Biopsy

Liquid biopsies, in which DNA sequencing can be performed on tumor components that are found circulating in the blood of cancer patients (including circulating tumor cells and cell-free circulating tumor DNA) have rapidly gained popularity in the past couple of years. These have given us a good opportunity to assess evolving tumor heterogeneity in real-time. These assays have proved to be highly sensitive and specific, with a high degree of concordance with tissue biopsy, they can identify both clonal and subclonal mutations, and they can detect resistance much earlier than radiographic imaging, which could permit earlier intervention, especially in lung cancer and hematolymphoid malignancies [32,33]. The first liquid biopsy-based companion diagnostic test was approved by the US Food and Drug Administration, in 2016, for the detection of EGFR mutations associated with NSCLC. A potential challenge with the application of ctDNA to NET absence of recurrent mutations in comparison with other tumors. Molecular profiling of small bowel NETs (SBNETs) revealed the most common recurrent mutations were in cyclin-dependent kinase inhibitor CDKN1B, occurring in only 8% of cases [34]. Pancreatic NETs (pNETs) are also characterized by recurrent mutations in a relatively limited number of genes, which include the tumor suppressor gene MEN1 as well as ATRX and DAXX, genes implicated in chromatin remodeling [35]. Yet, even liquid biopsy alone is not able to fully dissect the extent of tumor heterogeneity. Truly effective assessment of tumor heterogeneity is likely to require a combination of liquid biopsy, carefully selected tumor tissue biopsies, imaging diagnostics, and biomarkers. The main therapeutic strategies for overcoming tumor heterogeneity are focused on the mechanisms of resistance that it drives. It is becoming increasingly apparent that rationally designed combinations of drugs are likely to be required and might need to be administered early in the course of disease to prevent resistance. However, according to mathematical modeling studies, combinations of at least 3 drugs may be necessary [33]. An alternative strategy is to use checkpoint-inhibitor-based immunotherapy because a single treatment can target multiple neoantigens simultaneously. Although immunotherapy has proven to be a highly effective treatment paradigm in multiple tumor types, resistance still arises through varied mechanisms with tumor heterogeneity at their core.

### 3.5. Pharmacogenomics-Based Markers

**Figure 2:**
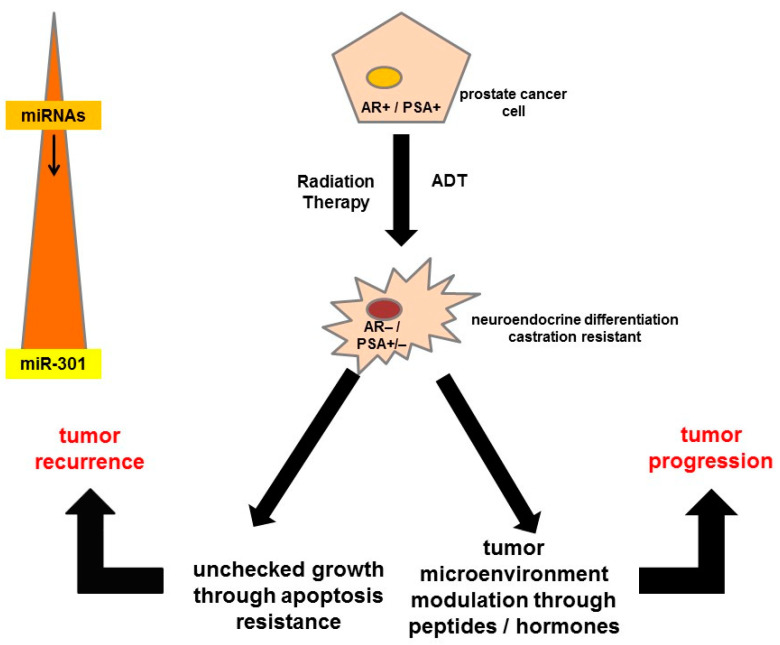
High heterogeneity among patients with metastasized CRPC after multiple different therapies at the stage when they are referred for Lu-177-PSMA therapies. The diagram gives a short overview of the different potentially activated signaling pathways on progression of prostate cancer cells that might become androgen-receptor- and prostate-specific antigen-negative over time, which can lead to apoptosis resistance and tumor recurrence or progression. ADT = androgen deprivation therapy, AR = androgen receptor, miRNA = microRNA, PSA = prostate-specific antigen.

The current Delphic consensus is that an accurate circulating biomarker that captures the biological activity of a NET and predicts its clinical behavior would provide an optimal method for the early detection of disease progression [36]. The NETest is a 51 multigene assay based on PCR analysis of specific NET circulating transcripts, and its results are depicted in the form of a score. It portrays the circulating NET fingerprints and exhibits a higher sensitivity and specificity (98 and 97%, respectively) than secretory markers for identifying neoplasia [37,38]. The assay is standardized and highly reproducible (inter- and intra-assay coefficient of variation <2%) and is postulated to be independent of tumor heterogeneity. Gene expression is captured in a 0–8 score derived from 4 different prediction algorithms that is mathematically scaled to disease activity (0–100%) by interpolating the expression of ‘omic’ transcripts that define specific biological components (hallmarks) of neoplasia. The clinical utility benefit has been documented in several independent clinical studies using diverse therapeutic strategies. In addition, the NETest has been demonstrated as an effective (85–90% accuracy) surrogate biomarker for tumor progression measured with conventional imaging with CT/MRI. A short PFS is significantly correlated (>95%) with increased blood biomarker levels > 40 (on a scale of 0–100). Similarly, RECIST progression of patients on somatostatin receptor binding analogs is also significantly associated (>90%) with increase in score. However, stabilization or response is associated with no change or reduction in scores (NETest levels ≤ 40). These alterations (progression NETest score > 40; disease stability ≤ 40) likely reflect the biological impact of treatment [39]. In neuroendocrine neoplasms of gastrointestinal tract, higher expression levels in tumor relative to non-tumor tissue of > tenfold were found for CgA, 2—tenfold higher mRNA levels were found for CD56, β-catenin, PDX1, CK20, and P53 and 1—twofold higher mRNA levels were found in CD45 tumor tissue compared with the non-tumor tissue [40]. A similar approach of using ‘omics’-based prognostication can also be used in prostate cancers, wherein the entire fulcrum of management is based on the biochemical value of PSA. Dysregulation of miRNAs (miRs) has been reported in prostate malignancy from early- to advanced stage and castration resistant disease progression [41]. Among differentially expressed miRNAs, most interestingly, the expression of miR-301 was upregulated in early-stage and CRPC progression, and this high expression of miR-301 was consistent in both serum and tumor tissue in prostate cancer patients compared to patients with benign prostate hyperplasia. In addition, miRNA regulatory genes during stage-specific prostate cancer progression suggest the involvement of p53, EGFR-PI3K-Akt, IGF, interleukins, TGFB, VEGF, JAK/STAT, WNT signaling and their effectors as the most critical genes in prostate cancer via upregulation of growth factor receptors, specifically EGFR, or through PTEN inactivation. An example of a patient with neuroendocrine differentiated prostate cancer is shown in Figure 2.

## 4. Principles of Radioligand Therapy

**Figure 3:**
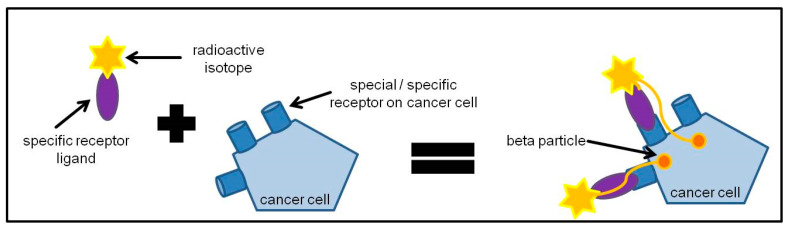
General principle of radioligand therapy. Cancer cells express special or sometimes specific receptors on their cell surface. Specific receptor ligands to these receptors can be labeled with a radioactive isotope that can emit beta particles. The specific receptor ligand binds to the receptors on the cancer cell and the beta particles come within close proximity to the cancer cell so that the cancer cell can be treated with beta particles and be killed.

A radioligand is made of two parts: a ligand, which can find cancer cells that have a particular surface molecule, and a radioisotope, which emits therapeutic radiation to kill these cells (Figure 3). The radioligand can target cells anywhere in the body. Radioligand binding is an approach that makes use of a radioactively labeled compound, which binds at the target binding site. These long-established assays are potentially suitable for purified protein, tissue homogenates, cell lysates, and also even whole cells. The radioligand tool compound should have a specific activity high enough to allow detection of the protein–ligand complex (usually by a scintillation counting method) in the binding assay and should demonstrate a high degree of selectivity and be pure and stable. The kinetics of the binding interaction are measured by detecting the incorporation of the radiolabeled peptides/antibodies over time. Radioligands should exhibit a high affinity for their targets, preferably having a dissociation rate constant (*K*d) in the subnanomolar to low nanomolar range. The ideal affinity for a radioligand depends on the expression level of the target receptor and should be at least 5- to 10-fold higher than the receptor expression (*B*max). High selectivity for its target is also required, preferably 100-fold less affinity for any other binding site which is expressed in the same level.

**Figure 4:**
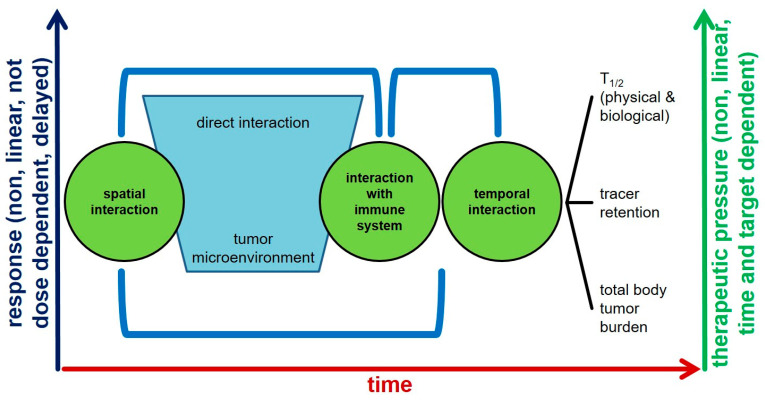
Radioligand therapy interaction with a cancer patient’s (closed) system. There are three ways in which a radioligand interacts with a tumor: (a) spatial interaction may be direct binding of the radioligand to the specific target on tumor or through interaction with tumor microenvironment e.g., vessels; (b) radioligand leads to modulation of tumor microenvironment to immune system and thus contributes to the efficacy of the treatment; and (c) temporal interaction due to differential physical and biological half-life, different rates of internationalization of the receptor–radioligand complex and also through the influence of amount of tumor burden in a patient as it directly influences the pharmacokinetics of the drug.

Moreover, the target-dependent model of radioligand therapy is the basic mechanism of localization. However, it has been seen that not all patients show response to treatment in spite of having the required number of receptors for binding of radioligands. Cancer cells change their responsiveness to drugs by changing their interaction with the surroundings. Earlier studies focused mainly on the cancer cell itself rather than on the interactions between the cancer cells and their surroundings. However, the role of tumor microenvironment (TME) in tumor progression and drug efficacy has recently attracted much attention. TME is the earliest determinant of ligand binding, and if multiple factors within the cancer cell such as immune response, hypoxic factors, etc., are not conductive to action of the protein–radionuclide complex, the further action is hampered, thereby affecting the therapeutic efficacy. Once the TME is favorable, the further course of action of radioligand is then determined by the physicochemical factors such as biological t1/2 and receptor density. This is a dynamic temporal process, as shown in Figure 4, and clarifies the concept of differential responses to radioligand therapy, in spite of the current judicious patient-selection-based criteria and guidelines.

In addition to the aforementioned factors, radioligand efficacy and toxicity also show a temporal relationship. Response of radioligand therapy is generally not dose dependent, is non-linear, and is delayed, sometimes manifesting itself 1–2 years after the last treatment cycle. On the other hand, the therapeutic pressure on the tumor biology is also non-linear. During treatment cycles, genetic alterations and the presence of different clones influences the degree of expression of targets, thereby directly influencing treatment efficacy.

### Radioligand Therapy and Tumor Heterogeneity

Radioligand therapy (RLT) focuses on targeting specific receptors on the surface of cancer cells. As a cancer progresses, there is an inherent heterogeneity that sets in and may lead to switching on and off of receptors. Selective receptor activation with changing tumor grade needs to be established objectively. This is possible with molecular imaging techniques; wherein receptor-specific radiopharmaceuticals provide us information about the degree of receptor expression. This receptor map can be further used as a guide for planning therapy using the same ligand which was used for imaging, by labeling it with a therapeutic radionuclide or, in some cases, targeted pharmacologic or chemotherapeutic agents can be used; this decision is based on the data supporting efficacy of therapy and toxicity profiles from the existing literature. 

Neuroendocrine tumors (NETs) include a heterogeneous group of malignancies arising in the diffuse neuroendocrine system and characterized by indolent growth. Complex interactions take place among the cellular components of the microenvironment of these tumors, and the recognition of the molecular mediators of their interplay and cross-talk is crucial to discovering novel therapeutic targets [42]. This heterogeneity presents a significant clinical challenge, a biopsy-proven WHO Grade 1 NET may sometimes behave aggressively, whereas at times, a Grade 3 tumor may demonstrate indolent behavior. Tissue biopsy has proven to be the ’tip of the iceberg’ as far as depiction of the cellular pattern of NETs are concerned. This clinical need has driven the search for accurate, affordable, and repeatable biomarkers to help inform prognosis and predict response to treatment. If identified, these biomarkers would allow administration of the right treatment to the right patient at the appropriate time—avoiding unnecessary side effects from therapy yet administering effective clinical treatment before significant clinical deterioration occurs. Apart from the prognostic implications, heterogeneity also makes it difficult to optimize treatment in NETs. 

**Figure 5:**
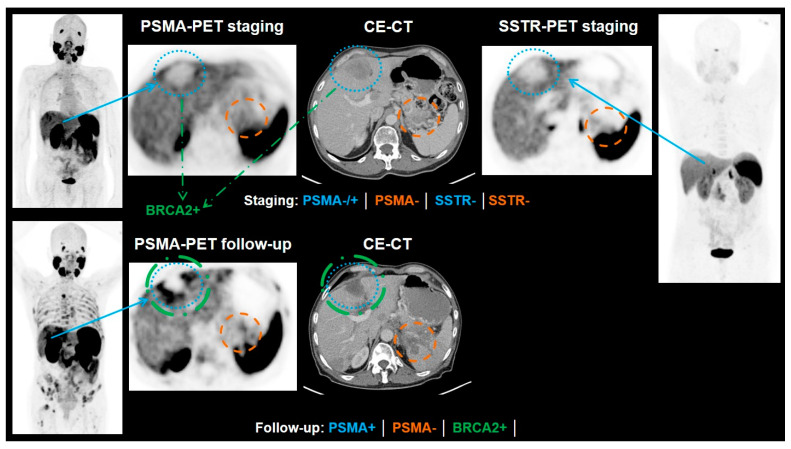
Example of a patient with neuroendocrine differentiated prostate cancer. At the time of the staging PSMA-PET, the patient had already tested positive for neuroendocrine differentiation of the prostate cancer. The PSA value had been below the detection rate. In the PSMA scan, the liver metastases showed only a PSMA expression similar to the liver uptake or only slightly above liver uptake (blue). A metastasis in the pancreas showed no PSMA expression (orange). An additional SSTR-PET was performed where no SSTR expression of the liver metastases (blue), nor was the pancreas metastasis (orange) detectable. After another biopsy of the marked liver metastasis, a BRCA2 mutation could be confirmed (green). The patient underwent chemotherapy with etoposid and cisplatin as well as an immunotherapy with olaparib. In the follow-up PSMA-PET, the patient presented with progressive disease, but the BRCA2-positive metastasis decreased in size (the lesion was additionally treated with Gamma Knife) and showed a higher PSMA expression than before (blue, green). In contrast, the metastasis of the pancreas increased in size and showed still no PSMA expression. CE-CT = contrast-enhancement computed tomography, PSMA = prostate-specific membrane antigen, SSTR = somatostatin receptor.

Radioligand therapy (RLT) targeting the prostate-specific membrane antigen (PSMA) is an emerging treatment modality for advanced prostate cancer, but 50% of patients with PSMA-positive tumors experience treatment failure. In tumors with different levels of PSMA expression for varying fractions of PSMA positive cells, PSMA expression correlates with radioligand uptake and DNA damage and, thus, RLT efficacy [43]. Intra- and interlesion variations in PSMA might result in undertreatment which reduces RLT efficacy and may select treatment resistant tumor clones [44]. One potential explanation for these discrepancies is heterogeneity of PSMA expression (Figure 4). Immunohistochemistry studies of mCRPC lesions have noted significant inter- and intrapatient heterogeneity of PSMA expression [45]. An example of heterogeneity in a case of prostate cancer is illustrated in Figure 5. Preclinical research has suggested that despite an overall increase in PSMA expression during progression of PCa from androgen sensitivity to androgen independence, some metastatic cell lines lose PSMA expression [46]. A significant proportion of liver metastases in CRPC patients may also lack PSMA expression [47]. Heterogeneity of PSMA expression may partly explain why about 30% of patients do not respond to 177Lu-PSMA RLT [48]. In contrast, low PSMA expression in patients with mCRPC who progress after conventional therapies may be a negative prognostic indicator [49]. 

## 5. Predictors of Response to Radioligand Therapy

Theranostics is the concept of patient selection for targeted radionuclide therapy based on the imaging phenotype on a companion diagnostic scan. However, emergence of heterogeneity in receptor expression across tumor grades has posed an imminent challenge toward administration of treatments based on standard recommendations and guidelines. There is an ‘unmet’ need to assign and implement targeted biomarkers which cover the entire gamut of heterogeneity that is known in respective tumors. In addition, predictive biomarkers, algorithms, and tools are the need of the hour, considering the cost of treatment, high specificity, and toxicity profiles of the newer therapies. Although conventional biomarkers are still used in clinics, such as serum chromogranin, 5-HIAA and neuron-specific enolase for NET, and serum PSA for prostate cancer, these fall short when tumors with mixed cellular patterns are presented to outpatient departments. We would therefore prefer to illustrate the ‘response predictors’ based on the various modalities used to assess treatment response.

### 5.1. Imaging Features and Target Heterogeneity

Imaging has several major advantages in assessing tumor heterogeneity over random tissue sampling, especially in patients with multiple metastases. First, it allows a full 3D volume assessment of the tumor and the analysis of different metastases from a same organ or from different organs. Secondly, it allows a longitudinal analysis over time. Finally, imaging enables to assess heterogeneity between patients with similar tumors (interpatient heterogeneity) as well as heterogeneity between different tumors within individual patient (intertumor heterogeneity) and within each lesion (intratumor heterogeneity). The analysis of spatial variation in architecture and/or function by imaging can provide benefit over simple biomarkers commonly used such as tumor size and average density measurement.

### 5.2. Vascular Heterogeneity

Due to their particular arterial supply, neuroendocrine liver metastases (NELM) are known to be hypervascular on arterial phase images and hypoattenuating on portal venous phase images in a majority of the patients (70–73%) [50,51,52,53]. Thus, dual phase CT (with a combination of arterial and portal venous phases) is mandatory for detection of NETs, both primary and metastatic [54]. However, about 30% of NELMs show a different enhancing pattern, including hypoenhancement of both arterial and portal phase in 12%, which explains the variable lesion conspicuity seen with dynamic phases imaging either with CT or MR. Finally, five different patterns of liver metastases from NET have been described: hypervascular, hypovascular, pseudocystic, pseudoangiomatous, and the military pattern [55,56,57]. The military pattern has been showed associated with higher risk of underestimation of lesion detection on CT and somatostatin analog imaging [54]. Recognition of different NET metastases enhancement patterns can have clinical consequences. First, it may be an indirect parameter in primary tumor identification. Indeed, significant differences in intratumoral vascularization have been shown depending on the primary tumor, as most enteric NET metastases (88%) showed a typical pattern, i.e., hypervascularity followed by washout on portal venous phase images, while this feature was only observed in 56% of pNEN metastases that are more frequently isointense on portal phase [50]. More interestingly, intratumoral vascular patterns were shown to be an imaging biomarker of tumor aggressiveness that is correlated with the histological grade of the tumor and the risk of metastases. On pathology, mean vascular density has been reported to be higher in well differentiated benign endocrine tumor, small lesions <2 cm, tumors with Ki-67 < 2%, non-metastatic tumors and in patients without disease progression [58]. Similar findings have been reported on CT and MR images where a well-circumscribed hypervascular mass with homogeneous enhancement is highly suggestive of a low-grade tumor. At the opposite end, an ill-defined hypovascular tumor on arterial and portal phase with heterogeneous enhancement is more common in grade 2 or NEC [59,60,61]. In agreement, quantitative assessment of tumors perfusion using dynamic contrast-enhanced CT technique (DCE-CT) has been shown to be significantly correlated with prognostic histological characteristics of pNEN [62]. Indeed, significant correlations existed between high blood flow and differentiation, proliferation index, or microvascular density on the one hand, and longer mean transit time and lymph node or liver metastases on the other hand. A link between blood flow and OS was also suggested but remains to be confirmed [62,63,64].

Vascular heterogeneity assessment is also a parameter of importance in selecting appropriate treatment of liver metastases. Indeed, intratumoral hypervascularization, defined as arterial contrast enhancement on imaging, has been shown to be a predictive factor of tumor response of hepatic intra-arterial therapy techniques such as transarterial chemoembolization (TACE) [64,65]. In addition to the qualitative assessment of enhancement on dual phase CT or MR images, a more reproducible quantitative approach with volumetric measurements of tumor enhancement could be easily performed using automatic segmentation of enhancing pixels. In general, volumetric analysis is applied on a lesion-by-lesion basis. However, this is impractical in most patients with NELM who present with multifocal, bilobar disease and are treated with lobar TACE. That is why some authors have proposed that volumetric assessment of the entire liver could be a more comprehensive biomarker for tumor response after TACE because it eliminates the subjectivity associated with lesion-based analysis and accounts for both tumor heterogeneity and tumor burden. Sahu et al. [66] evaluated the value of quantitative enhancing tumor burden in 51 patients with multifocal, bilobar NELM treated with TACE. The 50% cutoff in decrease of enhancement provided the best survival model. Moreover, the tumor burden enhancement response was the only biomarker associated with a survival difference between responders and non-responders and an independent predictor of survival (HR: 0.2; 95% CI: 0.1–0.6).

### 5.3. Cellularity Heterogeneity

Tumor cellularity heterogeneity can be assessed through imaging using diffusion weighted imaging (DWI) and the measured apparent diffusion coefficient (ADC), which can be performed quickly without need for the administration of contrast medium. Indeed, the diffusivity of water molecules is restricted in environments of high cellularity because this cellularity reduces the ratio of extracellular to intracellular space in a given area of tissue [67]. Studies conducted in vitro and in animal models show that the ADC is inversely correlated with tumor cellularity [68,69]. A quantitative assessment could be easily performed using the apparent diffusion coefficient (ADC) measuring signal attenuation being influenced by microscopic motion, including molecular diffusion of water as well as blood microcirculation. in several different types of tumors, the ADC value has been shown to be as a prognostic factor that can predict tumor grade [70,71].

On NETs, the addition of DWI sequences to morphological MRI revealed additional metastases and led to modifications of patient management. Adding DWI to standard liver MRI yielded additional findings for 45% of the patients with 1.78 times more new lesions, mainly infracentimetric; it induced a management change for 18% of the patients. DWI sequences added to whole-body MRI yielded additional findings for 71% of the patients, with 1.72 times more lesions, mainly infracentimetric, and induced a change in management for 19% of the patients [72]. Moreover, ADC values have been recently identified as a biomarker of tumor aggressiveness correlated with the histological grade on pNEN. Lotfalizadeh et al. found a significant inverse relation between ADC values and tumor grade, meaning that tumors with a higher grade showed lower ADC values when compared with those of lower grade. A cutoff of 1.19 × 10^3^ mm^2^/s was associated with a sensitivity of 100% and a specificity of 92% [73].

Another way to assess tumor cellularity in combination with tumor perfusion on MR imaging is the intravoxel incoherent motion diffusion weighted imaging (IVIM-DWI). First described in 1986 by Le Bihan et al. in neurologic disorders, IVIM is based on the fact that blood flow in capillaries mimics a diffusion process and impact diffusion MRI measurements [74]. Hence, the measurement of the ADC value on standard DW-MRI is biased by the effects of microcirculatory perfusion, which may impact the accuracy of ADC in evaluating hypervascular tumors such as NENs [75]. IVIM-based perfusion MRI, which does not require contrast agents but a specific acquisition of multiple b-value DWI and the analysis of the bi-exponential signal decay, has the potential to provide a single acquisition protocol for the non-invasive assessment of diffusion and perfusion in tissue. IVIM-DWI allows the measurement of two parameters, one representative of the tumor perfusion so-called fast apparent diffusion coefficient (D_fast_) and one representative of the tumor cellularity so-called slow apparent diffusion coefficient (D_slow_). IVIM-DWI has been used for differentiation of high-grade pancreatic ductal adenocarcinoma [76]. It was shown that high-grade pNEN had a significantly higher mean D_slow_ value and lower mean D_fast_ value in comparison to with pancreatic adenocarcinoma. Moreover, when D_slow_ and D_fast_ was combined, the specificity and sensitivity for differentiating high-grade pNENs from pancreatic adenocarcinoma were 77 and 100%, respectively. The translation of IVIM to clinical adoption requires DWI-MRI with shorter acquisition time, a consensus on the number and choice of b values and requirements for a sufficient signal-to-noise ratio level for accurate and reproducible post-processing.

### 5.4. Intratumoral Heterogeneity: The Radiomics Approach

Intratumor heterogeneity is near-ubiquitous in malignant tumors but varies between cancer and patients [77]. Qualitative analysis of intratumor heterogeneity and complexity is limited in morphological imaging, such as CT and MRI, and more often beyond the capability of human eyes. Radiomics is a new data-driven approach based on the assumption that images are a phenotype reflecting underlying biology and revealing more information than what the human eye would allow. Through dedicated software using mathematical statistical analysis of pixels or voxels of a region of interest, radiomics provide extraction of a large set of complex descriptors, so-called radiomics features [78]. Similarly to the genomic process, radiomics provide a radiological signature of the tumor with identification of radiomics features that could be correlated to outcome and could establish links between the imaging phenotype and genotypic and molecular characteristics of a tumor. The most common radiomics features include tumor size and shape, first order radiomics features based on the analysis of the voxel intensity histogram such as skewness, kurtosis, entropy, and more complex parameters from second or third order analysis based on special analysis of the relationship between voxels using a co-occurrence matrix and decomposition of the original image into low and high frequencies (Wavelet radiomics features).

Only few papers have addressed the usefulness of texture analysis and radiomics in NETs. Most of them focused on tumor grade prediction in pNEN [79,80,81,82,83,84]. The main characteristics and results of these studies are summarized in Table 1. In 4 of these 6 studies, only basic first order radiomics features were extracted from 2D ROI without independent validation. Kurtosis and entropy were the two radiomics features that seemed more relevant in predicting the pNEN grade. Two studies build a radiomics-based predictive model to non-invasively achieve pNEN grading using CT images. In both studies, the association of the radiomics signature (from either arterial or combined arterial + venous phase images) with clinical data had good accuracy in predicting the tumor grade of pNEN [80,83]. These promising results should not hide technical difficulties (influence of image acquisition parameters and reconstruction protocols, absence of harmonization of radiomics feature calculation methods) that still currently prevent the approach from being widely clinically used [84,85].

### 5.5. Tumor Proliferation Heterogeneity

Another well-known cause of heterogeneity of NENs is the tumor growth. Tumor growth is strongly correlated with the tumor proliferation index marker Ki-67, coming from the histopathological analysis of biopsy samples or surgical specimen, with low-Ki-67 tumors considered as slower-growing tumors compared to high-Ki-67 ones. However, this dichotomization between low-grade slow-growing and high-grade fast-growing tumors is not absolute since some histologic low-grade tumors may behave more like advanced progressive carcinomas. Moreover, grade 2 NEN tumors represent a very heterogeneous group of tumors with different growing profiles and aggressiveness potentials. Finally, tumors of different grades can be present in a NET patient at the same time. In addition to radionuclide imaging, conventional imaging has an important role in assessing tumor proliferation and potential heterogeneity between metastatic sites and between metastatic lesions.

In the past, several studies in metastatic GEP tumors showed a significant correlation between the tumor slope, assessed by tumor size measurement on consecutive CT examinations, and the patient outcome [86,87,88]. In these studies, the tumor slope was found to better reflect tumor aggressiveness than the disease-free interval or proliferative index. However, tumor slope assessment is not yet standardized in the field of endocrine tumors. Moreover, the period of time required for slope assessment delayed the prognostic classification and the treatment management as most authors consider there to be a low slope if the RECIST sum increases by <20% within one year. Another way to assess the spontaneous tumor growth is to measure the tumor growth rate (TGR), defined as an estimation of the increase/decrease of the tumor volume over time. TGR is expressed as the percentage change in tumor volume over one month. In post hoc analyses, tumor measurements from the CLARINET study (small bowel and pancreatic grade 1 and low grade 2 (Ki-67 > 10%) tumors) were re-evaluated to explore the clinical utility of TGR. A pretreatment TGR > 4%/month was associated with a 4.1-fold greater risk of progression than TGR ≤ 4%/month in the overall population (HR 4.1) [89]. The benefit of the treatment by lanreotide was also different depending on pretreatment TGR with intermediate TGR patients (between 4 and 10%) being those who responded best. TGR can be measured as an average for several metastatic lesions but can be also per lesion giving an opportunity to assess the in-patient heterogeneity between tumors. Such an approach, which allows identifying some metastatic lesions with a higher proliferation rate, could be of clinical value for the selection of treatments such as some local therapy. Moreover, one may hypothesize that incorporating metabolic imaging features known to be correlated with tumor grade, such as uptake on 68Ga-somatostatin receptor PET (SR-PET) and 18F-FDG-PET, may help to better cope with tumor heterogeneity.

Patients with prostate cancer are a heterogeneous group with different morbidity and mortality rates. Even with therapy, a significant proportion of patients show biochemical failure and subsequent metastatic progression, suggesting that additional strategies for personalized disease management are urgently needed. Intratumor heterogeneity (ITH) is considered to be one of the most important drivers of progression and resistance to therapy [90]. The heterogeneity of prostate cancer can, of course, also be assessed non-invasively using parameters from imaging with MRI. Radiomics application of multiparametric MRI in prostate cancer consist of high-resolution anatomic T2w sequences, diffusion weighted imaging (DWI), and dynamic contrast-enhanced sequences, and is occasionally supplemented by magnetic resonance spectroscopy. For local staging, prostate MRI is the most accurate imaging modality and is included in many national guidelines. However, most studies only include these 3 sequences (T2w, DWI, DCE) to build a model.

The applications for radiomics in prostate cancer [90,91,92,93,94,95,96,97,98,99] are summarized in Table 2. Of note, multiparametric MRI has so far only been studied for judgement of the prostatic gland/primary prostate cancer, but not for prostate cancer metastasis.

For NETs, MRI has been less frequently used for radiomics studies and has focused mainly on grading. In contrast to prostate cancer, MRI radiomics have been also applied to metastases and not only primary tumors. Table 3 shows the summary of applications and results.

In general, radiomics offers a cost-effective, non-invasive, and high-throughput approach in the analysis of image data, especially on MRI, which can lead to improved tumor detection and personalized treatment.

### 5.6. Future Directions

**Figure 6:**
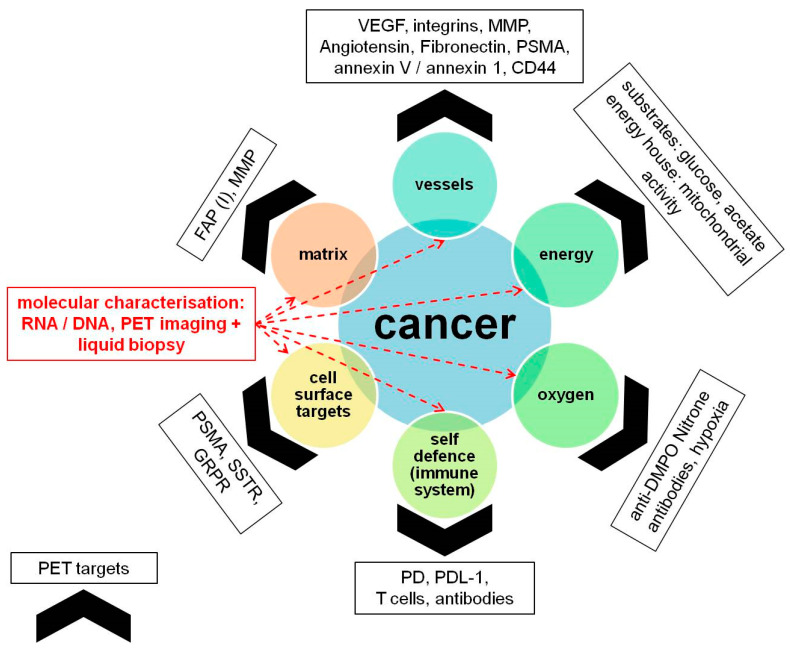
‘Territorial management’ of cancers. This concept involves using specific PET probes which identify the dominant pathways on tumor cells. The same ligands could then be used for targeting a therapeutic radionuclide for treatment of the cancers. Effective and ‘ideal’ therapy strategies should/must target all the elements simultaneously and monitor genetic mutations/signatures during therapy as well. CD44 = cluster of differentiation 44, DMPO = dimethylpyrrolineoxide, DNA = deoxyribonucleic acid, FAP (I) = fibroblast-activation-protein (inhibitors), GRPR = gastrin-releasing peptide receptor, MMP = matrix metalloproteinase, PD = programmed cell death protein, PDL-1 = programmed death-ligand 1, PSMA = prostate-specific membrane antigen, RNA = ribonucleic acid, SSTR = somatostatin receptor, VEGF = vascular endothelial growth factor.

The current landscape of theranostics and precision medicine revolves around identifying ‘targets’ which can be harnessed for imaging as well as therapy; its earliest example being use of iodine-131 for imaging and treatment of differentiated thyroid cancer. In times to follow, with the development of peptides, these sophisticated targets were used for neuroendocrine tumors and prostate cancer. However, resistance to these regimes have forced us to look at the tumor ‘territory’ from a different perspective. There are multiple pathways at play, and the need of the hour is to have a multi-pronged approach to overcome resistance. As illustrated in Figure 6, ‘territorial management’ of cancers involves using specific PET probes which identify the dominant pathways on tumor cells, and subsequent use of the same ligands for targeting therapeutic radionuclides (Table 4). Paschalis et al. [104] showed that deleterious DDR (DNA damage repair) aberrations are associated with replication stress, placing increased demand for metabolic precursors such as folate and glutamate, which are crucial to DNA synthesis and repair. As such, given the enzymatic capability of PSMA to yield glutamate and folate monoglutamate from polyglutamated folates and its reported role as a folate transporter, one possible explanation to account for this association is that PSMA overexpression in cells with deleterious DDR aberrations represents an adaptive cellular response. In this setting, PSMA overexpression may be driven by the increased requirement of these cells for cellular metabolites such as folate and glutamate. However, in all those patients who have shown non-response to PSMA RLT, there is a possibility that the tumor territory has another active pathway. Fibroblast activation protein (FAP) is a serine protease which is upregulated in several tumor types, while its expression in healthy adult tissues is scarce. FAP molecules and FAP+ stromal cells play an important although probably context-dependent and tumor type-specific pathogenetic role in tumor progression [105]. Kratochwil et al. [106]. have already used it as a target for imaging pancreas adenocarcinoma. These are the pathways which have been studied and elevated to clinical practice; however, the vast expanse of the tumor microenvironment provides us with even more opportunities to exploit several other pathways, e.g., with several PET tracers (Table 4), and account for possible ‘resistant’ cancer types, thereby improving outcomes.

Apart from utilizing the unique capability of PET tracers to map different receptors and metabolic/genetic pathways of tumors and their environment, there is also an urgent need for using state of the art tumor segmentation software for objectifying tumor heterogeneity. Khurshid et al. have shown the possibility of analyzing spatial heterogeneity of PSMA expression on mCRPC patients referred for Lu-177 PSMA therapy. A total of 328 bone, liver, and lymph node lesions from pretherapeutic PET/CT scans of 70 patients were analyzed using Interview Fusion Workstation (Mediso Medical Imaging System, Budapest, Hungary). The authors evaluated 5 heterogeneity parameters including entropy, contrast, size variation, homogeneity, and COV. The results were compared with change in PSA after RLT. Although not remarkably high, an area under curve of 0.695/0.683 for entropy/homogeneity showed promise for pre therapy image-based parameters for predicting response to RLT [107]. Similarly, entropy of pretherapy somatostatin receptor PET/CT was found to be an important prognostic marker for NET patients undergoing PRRT [108]. There are several other parameters such as kurtosis, skewness, solidity, etc., which can be gleamed out of PET images. However, validation of such parameters requires a significantly high number of patients. Therefore, it is equally important to integrate this information with other serum and tissue biomarkers to make a meaningful algorithm for individualized cancer treatment using artificial intelligence. This integration must go beyond any geopolitical boundaries, e.g., by establishing open image network platforms.

## Figures and Tables

**Table 1:** Published papers on the value of CT texture analysis in predicting the grade in pNEN.

	No of Patients	Images Phase/ROI/Sofware	Type RF	RF Correlated with G
D’Onofiro et al., 2019 [84], *Sci Rep*	100	Pancreatic 2D ROI 1 slice MaZda 4.6	1st order	Kurtosis and entropy
Guo et al., 2018 [79], *Abdo Imaging*	37	Arterial 2D Five slices Matlab 2014a	1st order	Mean grey level intensity
Canellas et al., 2018 [81], *AJR*	101	Portal TextRad 2D ROI	1st order	Entropy
Choi et al., 2018 [82], *Acta Radiol*	66	Arterial + portal 2D ROI	1st order	Sphericity, skewness, kurtosis
Gu et al., 2018 [80], *Eur Radiol*	104 training 34 validation	Arterial and portal 3D ROI Pyradiomics 1.3.0	1st, 2nd, and 3rd order radiomics signature: 15 arterial RF + 10 portal RF from 853 RF	Radiomics signature on arterial + portal images Nomagram: Radiomics + tumor margin
Liang et al., 2019 [83], *Clin Cancer Res*	86 training 51 validation	Arterial 3D ROI In-house software	1st, 2nd, and 3rd order radiomics signature 8RF from 467 RF	Monogram: Radiomics signature on arterial + clinical stage

MRI-based radiomics in prostate cancer und neuroendocrine tumors.

**Table 2:** Summary of radiomics literature in prostate cancer using MR.

Author	Study Type	Application	Number of Patients	Results
Fehr et al., 2015 [90]	Retrospective	Cancer risk prediction	217	Textural features from T2WI and ADC could distinguish between different Gleason scores. Accuracy of 93% after cross-validation for discrimination of Gleason 6 (3 + 3) vs. Gleason ≥ 7, and 92% for discrimination of Gleason 3 + 4 = 7a vs. 4 + 3 = 7b.
Woźnicki et al., 2020 [91]	Retrospective	Cancer risk prediction	191	Radiomics characterizes prostatic index lesions accurate and perform comparable to radiologists for prostate cancer characterization. Prognostic machine learning models could help in detection of clinically significant prostate cancer and patient selection for MRI-guided fusion biopsy.
Li et al., 2020 [92]	Retrospective	Cancer risk prediction	381	3 models were developed: a clinical model, a radiomics model (T2WI and ADC), and a clinical-radiomics combined model. Radiomic (AUC 0.98) and combined model (AUC 0.98) perform better in prediction of clinically significant cancer than clinical model (AUC 0.79)
Xu et al., 2019 [93]	Retrospective	Cancer risk prediction	331	6 selected radiomics features of MRI (T2WI and ADC) performed better (AUC 0.92) than each alone (T2WI: AUC 0.81, ADC: AUC 0.89). Individual preoperative prediction model performs better when including clinical factors and radiomic features (clinical model: AUC 0.73; combined model: AUC 0.93).
Ma et al., 2020 [94]	Retrospective	Staging	119	Radiomics signature based on 17 features on T2WIs has the potential to predict preoperative risk of extracapsular extension, good performance in the validation set (AUC 0.821).
Zhang et al., 2020 [95]	Retrospective	Tumor grading	166	Radiomics model with signatures from T2WI, ADC and DCE perform better than any single sequence (AUC: radiomics model 0.87; AUC T2WI/ADC/DCE: 0.70/0.76/0.73). Combined model with radiomics signature, clinical stage, and time from biopsy to RP outperformed the clinical model and radiomics model (AUC: combined model 0.91, clinical model 0.65, radiomics model 0.87). MpMRI had the potential to predict tumor upgrade from biopsy to RP.
Gnep et al., 2017 [96]	Retrospective	Therapy response Biochemical recurrence	74	T2WI Haralick textural features appear be strongly correlate with biochemical recurrence after radiotherapy.
Shiradkar et al., 2018 [97]	Retrospective	Therapy response Biochemical recurrence	120	10 extracted radiomic features from pretreatment T2WI and ADC are significantly correlated with BCR and could be used for BCR prediction; after radiotherapy?
Stoyanova et al., 2016 [98]	Retrospective	Radiogenomics	17	Radiomic features extracted from biopsy regions of primary tumors (?) and normal tissues correlate significant with gene signatures associated with adverse outcome.
Fischer et al., 2019 [99]	Retrospective	Radiogenomics	298	Biomarkers that play critical roles in PCa showed high correlation with aggressiveness-related imaging features extracted from mp-MRI images. The use of multi-omics data has the potential of significantly improving prediction of prostate cancer aggressiveness.

**Table 3:** Summary of radiomics manuscripts in neuroendocrine tumors related to MRI.

Author	Study Type	Application	Number of Patients	Results
Shi et al., 2020 [100]	Retrospective	Cancer risk prediction	66	Radiomics model based on diffusion kurtosis imaging (DKI) and T2 WI to discriminate pancreatic neuroendocrine tumors (PNETs) from solid pseudopapillary tumors (SPTs). 7 features of tumors were used to build radiomics model; the accuracy for diagnosis was higher than the radiologist (radiomics analysis 92.4%, radiologist 1 77.3%, radiologist 2 78.8%) and perform significantly better than of subjective diagnosis.
Bian et al., 2020 [101]	Retrospective	Tumor grading Primary or also mets?	157	7 final radiomic features was used for rad-score calculation. Rad-score correlate significantly with NF-pNET grades. This radiomic model could help to differentiate G1 and G2/3 non-invasive.
Guo et al., 2019 [102]	Retrospective	Tumor grading Primary or also mets?	77	Preoperative T2WI and DWI was used for texture feature extraction. AUC of best predicting model on T2WI was 0.99 (Grade 1 vs. Grade 3). This radiomic model could help to predict pNETs grading.
Weber et al., 2020 [103]	Retrospective	Therapy response Primary or also mets?	18	In this small sample size, no parameter from PET or ADC predicted treatment response to PRRT on pretherapeutic 68Ga-DOTATOC-PET/MRI. Treatment responder showed a significant decrease in lesion volume on ADC maps, no other textural feature from PET or ADC was statistically significant for differentiation between responders and non-responders.

**Table 4:** Overview of possible PET radiopharmaceuticals and their measured effects for functional imaging. AKT = serine/threonine protein kinase, CTLA-4 = cytotoxic T-lymphocyte-associated protein 4, CXCR-4 = chemokine receptor type 4, DNA = deoxyribonucleic acid, EGFR = epidermal growth factor receptor, FAPI = fibroblast-activation-protein inhibitors, GLP 1 = glucagon-like peptide 1, GRPR = gastrin-releasing peptide receptors, HER-2 = human epidermal growth factor receptor, PD-1 = programmed cell death protein, PDL-1 = programmed death-ligand 1, PIK3 = phosphoinositide 3-kinase, PSMA = prostate-specific membrane antigen.

PET Radiopharmaceuticals	Measured Effect
F-18 fluorodeoxyglucose	Aerobic and anaerobic glycolysis, glucose consumption or metabolism
C-11 thymidine, F-18 fluorothymidine	DNA synthesis, tumor cell proliferation
C-11 methionine	Protein synthesis, tumor cell proliferation
C-11 choline, F-18 fluorocholine	Cell-membrane metabolism, tumor-cell proliferation
C-11 tyrosine, F-18 fluorotyrosine, F-18 fluoroethyltyrosine	Natural amino acid transport
F-18 fluorodihydroxyphenylalanine	Dopamine synthesis, natural amino acid transport
F-18 fluoromisonidazole	Tissue hypoxia, identification of hypoxic tumor cells
F-18 fluoro-17-β-estradiol	Estrogen-receptor status
F-18 annexin V	Apoptotic cell death
F-18 fluorouracil	Accumulation of 5-fluorouracil in tumor
C-11 acetate	Lipid synthesis
F-18 siTATE, Ga-68 DOTA-X, Cu-64 DOTA-X In-111-octreotide, Ga-68 somatostatin receptor antagonists	Somatostatin receptor status
Ga-68/In-111 herceptin affibody	HER-2 receptor status
Ga-68 NODAGA RGD	Tumor neoangeogenesis
F-18 FEBM	EGFR expression
Ga-68 exendin 4	GLP 1 imaging
Ga-68 DOTA-mAB-F(ab’)2 cetuximab or HER3mAB105	Receptor tyrosine kinases; resistance to PI3K and AKT inhibitors
F-18 PSMA, Ga-68 PSMA	Prostate-specific membrane antigen
Zr-89 nivolumab, F-18 BMS 986192, Cu-64 pembrolizumab, C-64 ipilimumab, etc.	PD-1, PDL-1, CTLA-4
F-18, Ga-68-labeled FAPI	Tumor-associated fibroblast-activated protein
Ga-68 bombesin	Bombesin receptor, gastrin-releasing peptide receptors (GRPR)
Ga-68 pentixafor	CXCR-4

## References

[1] Hope T.A., Bodei L., Chan J.A., El-Haddad G., Fidelman N., Kunz P.L., Mailman J., Menda Y., Metz D.C., Mittra E.S. (2020). NANETS/SNMMI Consensus Statement on Patient Selection and Appropriate Use of 177Lu-DOTATATE Peptide Receptor Radionuclide Therapy. J. Nucl. Med..

[2] Strosberg J., El-Haddad G., Wolin E., Hendifar A., Yao J., Chasen B., Mittra E., Kunz P.L., Kulke M.H., Jacene H. (2017). Phase 3 Trial of 177Lu-Dotatate for Midgut Neuroendocrine Tumors. N. Engl. J. Med..

[3] McGranahan N., Swanton C. (2017). Clonal Heterogeneity and Tumor Evolution: Past, Present, and the Future. Cell.

[4] McGranahan N., Swanton C. (2015). Biological and therapeutic impact of intratumor heterogeneity in cancer evolution [published correction appears in Cancer Cell]. Cancer Cell.

[5] Von Bubnoff N. (2017). Liquid Biopsy: Approaches to Dynamic Genotyping in Cancer. Oncol. Res. Treat..

[6] Di Meo A., Bartlett J., Cheng Y., Pasic M.D., Yousef G.M. (2017). Liquid biopsy: A step forward towards precision medicine in urologic malignancies. Mol. Cancer.

[7] Dagogo-Jack I., Shaw A.T. (2018). Tumour heterogeneity and resistance to cancer therapies. Nat. Rev. Clin. Oncol..

[8] Wright N., Rida P.C.G., Aneja R. (2017). Tackling intra- and inter-tumor heterogeneity to combat triple negative breast cancer. Front. Biosci..

[9] Neuzillet C., Tijeras-Raballand A., Ragulan C., Cros J., Patil Y., Martinet M., Erkan M., Kleeff J., Wilson J., Apte M. (2019). Inter- and intra-tumoural heterogeneity in cancer-associated fibroblasts of human pancreatic ductal adenocarcinoma. J. Pathol..

[10] Prasetyanti P.R., Medema J.P. (2017). Intra-tumor heterogeneity from a cancer stem cell perspective. Mol. Cancer.

[11] O’Connor J.P., Rose C.J., Waterton J.C., Carano R.A., Parker G.J., Jackson A. (2015). Imaging Intratumor Heterogeneity: Role in Therapy Response, Resistance, and Clinical Outcome. Clin. Cancer Res..

[12] Walter D., Harter P.N., Battke F., Winkelmann R., Schneider M., Holzer K., Koch C., Bojunga J., Zeuzem S., Hansmann M.L. (2018). Genetic heterogeneity of primary lesion and metastasis in small intestine neuroendo-crine tumors. Sci Rep..

[13] Tolkach Y., Kristiansen G. (2018). The Heterogeneity of Prostate Cancer: A Practical Approach. Pathobiology.

[14] Salazar R., Wiedenmann B., Rindi G., Ruszniewski P. (2012). ENETS 2011 Consensus Guidelines for the Management of Patients with Digestive Neuroendocrine Tumors: An Update. Neuroendocrinology.

[15] Kvols L.K., Brendtro K.L. (2010). North American Neuroendocrine Tumor Society (NANETS). The North American Neuroendocrine Tumor Society (NANETS) guidelines: Mission, goals, and process. Pancreas.

[16] O’Toole D., Grossman A., Gross D., Fave G.D., Barkmanova J., Oconnor J.M., Pape U.-F., Plöckinger U. (2009). ENETS Consensus Guidelines for the Standards of Care in Neuroendocrine Tumors: Biochemical Markers. Neuroendocrinology.

[17] Papantoniou D., Grönberg M., Landerholm K., Welin S., Ziolkowska B., Nordvall D., Janson E.T. (2020). Assessment of hormonal levels as prognostic markers and of their optimal cut-offs in small intestinal neuroendocrine tumours grade 2. Endocrine.

[18] Modlin I.M., Gustafsson B.I., Moss S.F., Pavel M., Tsolakis A.V., Kidd M. (2010). Chromogranin A—Biological Function and Clinical Utility in Neuro Endocrine Tumor Disease. Ann. Surg. Oncol..

[19] Vezzosi D., Walter T., Laplanche A. (2011). Chromogranin A measurement in metastatic well-differentiated gastroentero-pancreatic neuroendocrine carcinoma: Screening for false positives and a prospective follow-up study. Int. J. Biol. Markers..

[20] Sang M., Hulsurkar M., Zhang X., Song H., Zheng D., Zhang Y., Li M., Xu J., Zhang S., Ittmann M. (2016). GRK3 is a direct target of CREB activation and regulates neuroendocrine differentiation of prostate cancer cells. Oncotarget.

[21] Li Y., Cozzi P.J. (2009). Angiogenesis as a strategic target for prostate cancer therapy. Med. Res. Rev..

[22] Desai H., Borges-Neto S., Wong T.Z. (2019). Molecular Imaging and Therapy for Neuroendocrine Tumors. Curr. Treat. Opt. Oncol..

[23] Hindié E. (2017). The NETPET Score: Combining FDG and Somatostatin Receptor Imaging for Optimal Management of Patients with Metastatic Well-Differentiated Neuroendocrine Tumors. Theranostics.

[24] Abdulrezzak U., Kurt Y.K., Kula M., Tutus A. (2016). Combined imaging with 68Ga-DOTA-TATE and 18F-FDG PET/CT on the basis of volumetric parameters in neuroendocrine tumors. Nucl. Med. Commun..

[25] Baum R.P., Prasad V., Cook G.J.R., Maisey M.N., Britton K.E., Chengazi A.V. (2006). “Monitoring Treatment,” in Clinical Nuclear Medicine.

[26] Graf J., Pape U.F., Jann H., Denecke T., Arsenic R., Brenner W., Pavel M., Prasad V. (2020). Prognostic Significance of Somatostatin Receptor Heterogeneity in Progressive Neuroendo-crine Tumor Treated with Lu-177 DOTATOC or Lu-177 DOTATATE. Eur. J. Nucl. Med. Mol. Imaging.

[27] Farolfi A., Fendler W., Iravani A., Haberkorn U., Hicks R., Herrmann K., Walz J., Fanti S. (2019). Theranostics for Advanced Prostate Cancer: Current Indications and Future Developments. Eur. Urol. Oncol..

[28] Ravi Kumar A.S., Hofman M.S. (2020). Mechanistic Insights for Optimizing PSMA Radioligand Therapy. Clin Cancer Res..

[29] Hofman M.S., Violet J., Hicks R.J., Ferdinandus J., Thang S.P., Akhurst T., Iravani A., Kong G., Kumar A.R., Murphy D.G. (2018). [177 Lu]-PSMA-617 radionuclide treatment in patients with metastatic castration-resistant prostate cancer (LuPSMA trial): A single-centre, single-arm, phase 2 study. Lancet Oncol..

[30] Thang S.P., Violet J., Sandhu S., Iravani A., Akhurst T., Kong G., Hofman M.S. (2019). Poor Outcomes for Patients with Metastatic Castration-resistant Prostate Cancer with Low Prostate-specific Membrane Antigen (PSMA) Expression Deemed Ineligible for 177Lu-labelled PSMA Radioligand Ther-apy. Eur. Urol. Oncol..

[31] Schmidkonz C., Cordes M., Schmidt D., Bäuerle T., Goetz T.I., Beck M., Prante O., Cavallaro A., Uder M., Wullich B. (2018). 68Ga-PSMA-11 PET/CT-derived metabolic parameters for determination of whole-body tumor burden and treatment response in prostate cancer. Eur. J. Nucl. Med. Mol. Imaging.

[32] Cheng F., Su L., Qian C. (2016). Circulating tumor DNA: A promising biomarker in the liquid biopsy of cancer. Oncotarget.

[33] Moreno J.G., Gomella L.G. (2019). Evolution of the Liquid Biopsy in Metastatic Prostate Cancer. Urology.

[34] Francis J.M., Kiezun A., Ramos A.H., Serra S., Pedamallu C.S., Qian Z.R., Banck M.S., Kanwar R., A Kulkarni A., Karpathakis A. (2013). Somatic mutation of CDKN1B in small intestine neuroendocrine tumors. Nat. Genet..

[35] Jiao Y., Shi C., Edil B.H., De Wilde R.F., Klimstra D.S., Maitra A., Papadopoulos N. (2011). DAXX/ATRX, MEN1, and mTOR pathway genes are frequently altered in pancreatic neuroendo-crine tumors. Science.

[36] Oberg K., Modlin I., DeHerder W., Pavel M., Klimstra D., Frilling A., Metz D., Heaney A., Kwekkeboom D., Strosberg J. (2015). Biomarkers for neuroendocrine tumor disease: A delphic consensus assessment of multianalytes, genomics, circulating cells and monoanalytes. Lancet Oncol..

[37] Modlin I.M., Kidd M., Malczewska A., Drozdov I., Bodei L., Matar S., Chung K.M. (2018). The NETest: The Clinical Utility of Multigene Blood Analysis in the Diagnosis and Management of Neuroendocrine Tumors. Endocrinol. Metab. Clin. N. Am..

[38] Pavel M., Jann H., Prasad V., Drozdov I., Modlin I.M., Kidd M. (2017). NET Blood Transcript Analysis Defines the Crossing of the Clin-ical Rubicon: When Stable Disease Becomes Progressive. Neuroendocrinology.

[39] Bodei L., Kidd M.S., Singh A., Van Der Zwan W.A., Severi S., Drozdov I., Cwikla J.B., Baum R.P., Kwekkeboom D.J., Paganelli G. (2018). PRRT genomic signature in blood for prediction of 177Lu-octreotate efficacy. Eur. J. Nucl. Med. Mol. Imaging.

[40] Kyriakopoulos G., Mavroeidi V., Chatzellis E., Kaltsas G.A., Alexandraki K.I. (2018). Histopathological, immunohistochemical, genet-ic and molecular markers of neuroendocrine neoplasms. Ann. Transl. Med..

[41] Balázs K., Antal L., Sáfrány G., Lumniczky K. (2021). Blood-Derived Biomarkers of Diagnosis, Prognosis and Therapy Response in Prostate Cancer Patients. J. Pers. Med..

[42] Oberg K., Akerström G., Rindi G., Jelic S., ESMO Guidelines Working Group (2010). Neuroendocrine gastroenteropancreatic tu-mours: ESMO Clinical Practice Guidelines for diagnosis, treatment and follow-up. Ann. Oncol..

[43] Virgolini I., Traub T., Novotny C., Leimer M., Füger B., Li S., Patri P., Pangerl T., Angelberger P., Raderer M. (2001). New trends in peptide receptor radioligands. Q. J. Nucl. Med. Off. Publ. Ital. Assoc. Nucl. Med. (AIMN)/Int. Assoc. Radiopharm. (IAR).

[44] Baum R.P., Kulkarni H.R., Schuchardt C., Singh A., Wirtz M., Wiessalla S., Schottelius M., Mueller D., Klette I., Wester H.-J. (2016). 177Lu-Labeled Prostate-Specific Membrane Antigen Radioligand Therapy of Metastatic Castration-Resistant Prostate Cancer: Safety and Efficacy. J. Nucl. Med..

[45] Bahmad H.F., Jalloul M., Azar J., Moubarak M.M., Samad T.A., Mukherji D., Al-Sayegh M., Abou-Kheir W. (2021). Tumor Microenviron-ment in Prostate Cancer: Toward Identification of Novel Molecular Biomarkers for Diagnosis, Prognosis, and Therapy De-velopment. Front Genet..

[46] Lückerath K., Wei L., Fendler W.P., Axelsson S.E., Stuparu A.D., Slavik R., Mona C.E., Calais J., Rettig M., Reiter R.E. (2018). Preclinical evaluation of PSMA expression in response to androgen receptor blockade for theranostics in prostate cancer. EJNMMI Res..

[47] Khreish F., Kochems N., Rosar F., Sabet A., Ries M., Maus S., Ezziddin S. (2020). Response and outcome of liver metastases in patients with metastatic castration-resistant prostate cancer (mCRPC) undergoing 177Lu-PSMA-617 radioligand therapy. Eur. J. Nucl. Med. Mol. Imaging.

[48] Ruigrok E.A.M., van Weerden W.M., Nonnekens J., de Jong M. (2019). The Future of PSMA-Targeted Radionuclide Therapy: An Over-view of Recent Preclinical Research. Pharmaceutics.

[49] Current K., Meyer C., Magyar C.E., Mona C.E., Almajano J., Slavik R., Stuparu A.D., Cheng C., Dawson D.W., Radu C.G. (2020). Investigating PSMA-Targeted Radioligand Therapy Efficacy as a Function of Cellular PSMA Levels and Intratumoral PSMA Heterogeneity. Clin. Cancer Res..

[50] Ronot M., Cuccioli F., Burgio M.D., Vullierme M.-P., Hentic O., Ruszniewski P., D’Assignies G., Vilgrain V. (2017). Neuroendocrine liver metastases: Vascular patterns on triple-phase MDCT are indicative of primary tumour location. Eur. J. Radiol..

[51] Dromain C., De Baere T., Lumbroso J., Caillet H., Laplanche A., Boige V., Ducreux M., Duvillard P., Elias D., Schlumberger M. (2005). Detection of Liver Metastases from Endocrine Tumors: A Prospective Comparison of Somatostatin Receptor Scintigraphy, Computed Tomography, and Magnetic Resonance Imaging. J. Clin. Oncol..

[52] Paulson E.K., McDermott V.G., Keogan M.T., Delong D.M., Frederick M.G., Nelson R.C. (1998). Carcinoid metastases to the liver: Role of triple-phase helical CT. Radiology.

[53] Foley W.D., Mallisee T.A., Hohenwalter M.D., Wilson C.R., Quiroz F.A., Taylor A.J. (2000). Multiphase hepatic CT with a multirow detector CT scanner. AJR Am. J. Roentgenol..

[54] Oliver J.H., Baron R.L., Federle M.P., Jones B.C., Sheng R. (1997). Hypervascular liver metastases: Do unenhanced and hepatic arterial phase CT images affect tumor detection?. Radiology.

[55] Dromain C., De Baere T., Baudin E., Galline J., Ducreux M., Boige V., Duvillard P., Laplanche A., Caillet H., Lasser P. (2003). MR Imaging of Hepatic Metastases Caused by Neuroendocrine Tumors: Comparing Four Techniques. Am. J. Roentgenol..

[56] Elias D., Lefevre J.H., Duvillard P., Goéré D., Dromain C., Dumont F., Baudin E. (2010). Hepatic metastases from neuroendocrine tumors with a “thin slice” pathological ex-amination: They are many more than you think. Ann. Surg..

[57] Soyer P., Gueye C., Somveille E., Laissy J.P., Scherrer A. (1995). MR diagnosis of hepatic metastases from neuroendocrine tumors versus hemangio-mas: Relative merits of dynamic gadolinium chelate-enhanced gradient-recalled echo and unenhanced spin-echo images. AJR Am. J. Roentgenol..

[58] Marion-Audibert A.M., Barel C., Gouysse G., Dumortier J., Pilleul F., Pourreyron C., Scoazec J.Y. (2003). Low microvessel density is an unfavorable histoprognostic factor in pan-creatic endocrine tumors. Gastroenterology.

[59] Takumi K., Fukukura Y., Higashi M., Ideue J., Umanodan T., Hakamada H., Kanetsuki I., Yoshiura T. (2015). Pancreatic neuroendocrine tumors: Correlation between the contrast-enhanced computed tomography features and the pathological tumor grade. Eur. J. Radiol..

[60] Kim J.H., Eun H.W., Kim Y.J., Han J.K., Choi B.I. (2013). Staging accuracy of MR for pancreatic neuroendocrine tumor and imaging findings accord-ing to the tumor grade. Abdom. Imaging.

[61] De Robertis R., Cingarlini S., Martini P.T., Ortolani S., Butturini G., Landoni L., D’Onofrio M. (2017). Pancreatic neuroendocrine neoplasms: Magnetic resonance imaging features according to grade and stage. World J. Gastroenterol..

[62] Rodallec M., Vilgrain V., Couvelard A., Rufat P., O’Toole D., Barrau V., Menu Y. (2006). Endocrine pancreatic tumours and helical CT: Contrast enhancement is correlat-ed with microvascular density, histoprognostic factors and survival. Pancreatology.

[63] d’Assignies G., Couvelard A., Bahrami S., Vullierme M.P., Hammel P., Hentic O., Vilgrain V. (2009). Pancreatic Endocrine Tumors: Tumor Blood Flow Assessed with Perfusion CT Reflects Angiogenesis and Correlates with Prognostic Factors. Radiology.

[64] Marrache F., Vullierme M.P., Roy C., El Assoued Y., Couvelard A., O’Toole D., Mitry E., Hentic O., Hammel P., Lévy P. (2006). Arterial phase enhancement and body mass index are predictors of response to chemoembolisation for liver metastases of endocrine tumours. Br. J. Cancer.

[65] Roche A., Girish B.V., De Baere T., Ducreux M., Elias D., Laplanche A., Boige V., Schlumberger M., Ruffle P., Baudin E. (2004). Prognostic factors for chemoembolization in liver metastasis from endocrine tumors. Hepatogastroenterology.

[66] Sahu S., Schernthaner R., Ardon R., Chapiro J., Zhao Y., Sohn J.H., Duran R. (2017). Imaging Biomarkers of Tumor Response in Neuroendocrine Liver Metastases Treat-ed with Transarterial Chemoembolization: Can Enhancing Tumor Burden of the Whole Liver Help Predict Patient Survival?. Radiology.

[67] Koh D.-M., Collins D. (2007). Diffusion-Weighted MRI in the Body: Applications and Challenges in Oncology. Am. J. Roentgenol..

[68] Kuroda M., Matsumoto Y., Matsuya R., Kato H., Shibuya K., Oita M., Kawabe A., Matsuzaki H., Asaumi J., Murakami J. (2009). In vitro experimental study of the relationship between the apparent diffusion coefficient and changes in cellularity and cell morphology. Oncol. Rep..

[69] Lyng H., Haraldseth O., Rofstad E.K. (2000). Measurement of cell density and necrotic fraction in human melanoma xenografts by diffusion weighted magnetic resonance imaging. Magn. Reson. Med..

[70] Yoshikawa M.I., Ohsumi S., Sugata S., Kataoka M., Takashima S., Mochizuki T., Ikura H., Imai Y. (2008). Relation between cancer cellularity and apparent diffusion coefficient values using diffusion-weighted magnetic resonance imaging in breast cancer. Radiat. Med..

[71] Chen L., Liu M., Bao J., Xia Y., Zhang J., Zhang L., Huang X., Wang J. (2013). The Correlation between Apparent Diffusion Coefficient and Tumor Cellularity in Patients: A Meta-Analysis. PLoS ONE.

[72] d’Assignies G., Fina P., Bruno O., Vullierme M.P., Tubach F., Paradis V., Vilgrain V. (2013). High sensitivity of diffusion-weighted MR imaging for the detection of liver metasta-ses from neuroendocrine tumors: Comparison with T2-weighted and dynamic gadolinium-enhanced MR imaging. Radiology.

[73] Lotfalizadeh E., Ronot M., Wagner M., Cros J., Couvelard A., Vullierme M.P., Vilgrain V. (2016). Prediction of pancreatic neuroendocrine tumour grade with MR imaging fea-tures: Added value of diffusion-weighted imaging. Eur. Radiol..

[74] Le Bihan D., Breton E., Lallemand D., Grenier P., Cabanis E., Laval-Jeantet M. (1986). MR imaging of intravoxel incoherent motions: Application to diffusion and perfu-sion in neurologic disorders. Radiology.

[75] Chandarana H., Lee V.S., Hecht E., Taouli B., Sigmund E.E. (2011). Comparison of biexponential and monoexponential model of diffusion weighted imaging in evaluation of renal lesions: Preliminary experience. Investig. Radiol..

[76] Ma W., Wei M., Han Z., Tang Y., Pan Q., Zhang G., Ren J., Huan Y., Li N. (2019). The added value of intravoxel incoherent motion diffusion weighted imaging parameters in differentiating high-grade pancreatic neuroendocrine neoplasms from pancreatic ductal adenocarcinoma. Oncol. Lett..

[77] Meacham C.E., Morrison S.J. (2013). Tumour heterogeneity and cancer cell plasticity. Nature.

[78] Limkin E.J., Sun R., Dercle L., Zacharaki E.I., Robert C., Reuzé S., Ferté C. (2017). Promises and challenges for the implementation of computational medical imaging (radi-omics) in oncology. Ann. Oncol..

[79] Guo C., Zhuge X., Wang Z., Wang Q., Sun K., Feng Z., Chen X. (2019). Textural analysis on contrast-enhanced CT in pancreatic neuroendocrine neoplasms: Associa-tion with WHO grade. Abdom. Radiol..

[80] Gu D., Hu Y., Ding H., Wei J., Chen K., Liu H., Zeng M., Tian J. (2019). CT radiomics may predict the grade of pancreatic neuroendocrine tumors: A multicenter study. Eur. Radiol..

[81] Canellas R., Burk K.S., Parakh A., Sahani D.V. (2018). Prediction of Pancreatic Neuroendocrine Tumor Grade Based on CT Features and Tex-ture Analysis. AJR Am. J. Roentgenol..

[82] Choi T.W., Kim J.H., Yu M.H., Park S.J., Han J.K. (2017). Pancreatic neuroendocrine tumor: Prediction of the tumor grade using CT findings and computerized texture analysis. Acta Radiol..

[83] Liang W., Yang P., Huang R., Xu L., Wang J., Liu W., Zhang L., Wan D., Huang Q., Lu Y. (2019). A Combined Nomogram Model to Preoperatively Predict Histologic Grade in Pancreatic Neuroendocrine Tumors. Clin. Cancer Res..

[84] D’Onofrio M., Ciaravino V., Cardobi N., De Robertis R., Cingarlini S., Landoni L., Scarpa A. (2019). CT Enhancement and 3D Texture Analysis of Pancreatic Neuroendocrine Neo-plasms. Sci. Rep..

[85] Reuzé S., Schernberg A., Orlhac F., Sun R., Chargari C., Dercle L., Deutsch E., Buvat I., Robert C. (2018). Radiomics in Nuclear Medicine Applied to Radiation Therapy: Methods, Pitfalls, and Challenges. Int. J. Radiat. Oncol..

[86] Durante C., Boukheris H., Dromain C., Duvillard P., Leboulleux S., Elias D., Baudin E. (2009). Prognostic factors influencing survival from metastatic (stage IV) gastroentero-pancreatic well-differentiated endocrine carcinoma. Endocr. Relat. Cancer.

[87] Palazzo M., Lombard-Bohas C., Cadiot G., Matysiak-Budnik T., Rebours V., Vullierme M.-P., Couvelard A., Hentic O., Ruszniewski P. (2013). Ki67 proliferation index, hepatic tumor load, and pretreatment tumor growth predict the antitumoral efficacy of lanreotide in patients with malignant digestive neuroendocrine tumors. Eur. J. Gastroenterol. Hepatol..

[88] Madeira I., Terris B., Voss M., Denys A., Sauvanet A., Flejou J.-F., Vilgrain V., Belghiti J., Bernades P., Ruszniewski P. (1998). Prognostic factors in patients with endocrine tumours of the duodenopancreatic area. Gut.

[89] Dromain C., Pavel M.E., Ruszniewski P., Langley A., Massien C., Baudin E., Caplin M.E. (2019). Tumor growth rate as a metric of progression, response, and prognosis in pan-creatic and intestinal neuroendocrine tumors. BMC Cancer.

[90] Fehr D., Veeraraghavan H., Wibmer A.G., Gondo T., Matsumoto K., Vargas H.A., Sala E., Hricak H., Deasy J. (2015). Automatic classification of prostate cancer Gleason scores from multiparametric magnetic resonance images. Proc. Natl. Acad. Sci. USA.

[91] Woźnicki P., Westhoff N., Huber T., Riffel P., Froelich M.F., Gresser E., Nörenberg D. (2020). Multiparametric MRI for Prostate Cancer Characterization: Combined Use of Radi-omics Model with PI-RADS and Clinical Parameters. Cancers.

[92] Li M., Chen T., Zhao W., Wei C., Li X., Duan S., Ji L., Lu Z., Shen J. (2020). Radiomics prediction model for the improved diagnosis of clinically significant prostate cancer on biparametric MRI. Quant. Imaging Med. Surg..

[93] Xu M., Fang M., Zou J., Yang S., Yu D., Zhong L., Hu C., Zang Y., Dong D., Tian J. (2019). Using biparametric MRI radiomics signature to differentiate between benign and malignant prostate lesions. Eur. J. Radiol..

[94] Ma S., Xie H., Wang H., Yang J., Han C., Wang X., Zhang X. (2019). Preoperative Prediction of Extracapsular Extension: Radiomics Signature Based on Magnetic Resonance Imaging to Stage Prostate Cancer. Mol. Imaging Biol..

[95] Zhang G., Han Y., Wei J., Qi Y., Gu D., Lei J., Yan W., Xiao Y., Xue H., Feng F. (2020). Radiomics Based on MRI as a Biomarker to Guide Therapy by Predicting Upgrading of Prostate Cancer from Biopsy to Radical Prostatectomy. J. Magn. Reson. Imaging.

[96] Gnep K., Fargeas A., Gutiérrez-Carvajal R.E., Commandeur F., Mathieu R., Ospina J.D., de Crevoisier R. (2017). Haralick textural features on T(2)-weighted MRI are associated with bio-chemical recurrence following radiotherapy for peripheral zone prostate cancer. J. Magn. Reson. Imaging.

[97] Shiradkar R., Ghose S., Jambor I., Taimen P., Ettala O., Purysko A.S., Madabhushi A. (2018). Radiomic features from pretreatment biparametric MRI predict prostate cancer bio-chemical recurrence: Preliminary findings. J. Magn. Reson. Imaging.

[98] Stoyanova R., Pollack A., Takhar M., Lynne C., Parra N., Lam L.L., Alshalalfa M., Buerki C., Castillo R., Jorda M. (2016). Association of multiparametric MRI quantitative imaging features with prostate cancer gene expression in MRI-targeted prostate biopsies. Oncotarget.

[99] Fischer S., Tahoun M., Klaan B., Thierfelder K.M., Weber M.-A., Krause B.J., Hakenberg O., Fuellen G., Hamed M. (2019). A Radiogenomic Approach for Decoding Molecular Mechanisms Underlying Tumor Progression in Prostate Cancer. Cancers.

[100] Shi Y.J., Zhu H.T., Liu Y.L., Wei Y.Y., Qin X.B., Zhang X.Y., Sun Y.S. (2020). Radiomics Analysis Based on Diffusion Kurtosis Imaging and T2 Weighted Imaging for Differ-entiation of Pancreatic Neuroendocrine Tumors from Solid Pseudopapillary Tumors. Front. Oncol..

[101] Bian Y., Li J., Cao K., Fang X., Jiang H., Ma C., Wang L. (2020). Magnetic resonance imaging radiomic analysis can preoperatively predict G1 and G2/3 grades in patients with NF-pNETs. Abdom. Radiol..

[102] Guo C.-G., Ren S., Chen X., Wang Q.-D., Xiao W.-B., Zhang J.-F., Duan S.-F., Wang Z.-Q. (2019). Pancreatic neuroendocrine tumor: Prediction of the tumor grade using magnetic resonance imaging findings and texture analysis with 3-T magnetic resonance. Cancer Manag. Res..

[103] Weber M., Kessler L., Schaarschmidt B., Fendler W.P., Lahner H., Antoch G., Rischpler C. (2020). Treatment-related changes in neuroendocrine tumors as assessed by textural features derived from (68)Ga-DOTATOC PET/MRI with simultaneous acquisition of apparent diffusion coefficient. BMC Cancer.

[104] Paschalis A., Sheehan B., Riisnaes R., Rodrigues D.N., Gurel B., Bertan C., Ferreira A., Lambros M.B., Seed G., Yuan W. (2019). Prostate-specific Membrane Antigen Heterogeneity and DNA Repair Defects in Prostate Cancer. Eur. Urol..

[105] Busek P., Mateu R., Zubal M., Kotackova L., Sedo A. (2018). Targeting fibroblast activation protein in cancer—Prospects and caveats. Front. Biosci..

[106] Kratochwil C., Flechsig P., Lindner T., Abderrahim L., Altmann A., Mier W., Adeberg S., Rathke H., Röhrich M., Winter H. (2019). 68Ga-FAPI PET/CT: Tracer Uptake in 28 Different Kinds of Cancer. J. Nucl. Med..

[107] Khurshid Z., Ahmadzadehfar H., Gaertner F.C., Papp L., Zsóter N., Essler M., Bundschuh R.A. (2018). Role of textural heterogeneity parameters in patient selection for 177Lu-PSMA therapy via response prediction. Oncotarget.

[108] Werner R.A., Lapa C., Ilhan H., Higuchi T., Buck A.K., Lehner S., Bartenstein P., Bengel F., Schatka I., Muegge D.O. (2017). Survival prediction in patients undergoing radionuclide therapy based on in-tratumoral somatostatin-receptor heterogeneity. Oncotarget.

